# Studies in the mouse model identify strain variability as a major determinant of disease outcome in *Leishmania infantum* infection

**DOI:** 10.1186/s13071-015-1259-6

**Published:** 2015-12-18

**Authors:** Filipe Marques, Sílvia Vale-Costa, Tânia Cruz, Joana Moreira Marques, Tânia Silva, João Vilares Neves, Sofia Cortes, Ana Fernandes, Eduardo Rocha, Rui Appelberg, Pedro Rodrigues, Ana M. Tomás, Maria Salomé Gomes

**Affiliations:** Instituto de Investigação e Inovação em Saúde and IBMC, Instituto de Biologia Molecular e Celular, Universidade do Porto, Porto, Portugal; Present address: Cell Biology of Viral Infection Lab, Instituto Gulbenkian de Ciência, Oeiras, Portugal; Instituto de Investigação e Inovação em Saúde; IBMC, Instituto de Biologia Molecular e Celular, and ICBAS, Instituto de Ciências Biomédicas Abel Salazar, Universidade do Porto, Porto, Portugal; GHTM, Global Health and Tropical Medicine, IHMT, Instituto de Higiene e Medicina Tropical, Universidade Nova de Lisboa, Lisbon, Portugal; Present Address: Molekularbiologie und Funktionelle Genomik, Technische Hochschule Wildau, Wildau, Germany; ICBAS, Instituto de Ciências Biomédicas Abel Salazar, Universidade do Porto, Porto, Portugal; ICBAS, Instituto de Ciências Biomédicas Abel Salazar, and CIIMAR, Centro Interdisciplinar de Investigação Marinha e Ambiental, Universidade do Porto, Porto, Portugal

## Abstract

**Background:**

Visceral leishmaniasis is a severe and potentially fatal disease caused by protozoa of the genus *Leishmania*, transmitted by phlebotomine sandflies. In Europe and the Mediterranean region, *L. infantum* is the commonest agent of visceral leishmaniasis, causing a wide spectrum of clinical manifestations, including asymptomatic carriage, cutaneous lesions and severe visceral disease. Visceral leishmaniasis is more frequent in immunocompromised individuals and data obtained in experimental models of infection have highlighted the importance of the host immune response, namely the efficient activation of host’s macrophages, in determining infection outcome. Conversely, few studies have addressed a possible contribution of parasite variability to this outcome.

**Methods:**

In this study, we compared three isolates of *L. infantum* regarding their capacity to grow in the organs of mice, the way they activate the host’s macrophages and other components of the immune response and also their capacity to cope with host’s antimicrobial mechanisms, namely reactive oxygen and nitrogen species.

**Results:**

We found that the three parasite strains significantly differed regarding the degree to which they induced nitric oxide synthase (NOS2) and arginase expression in infected macrophages and the pattern of cytokine production they induced in the host, resulting in different degrees of inflammatory response in infected livers. Additionally, the three strains also significantly differed in their in vitro susceptibility to reactive oxygen and nitrogen species. This variability was reflected in the capacity of each strain to persist and proliferate in the organs of wild-type as well as NOS2- and phagocyte oxidase- deficient mice.

**Conclusions:**

The results obtained in this study show that parasite strain variability is an important determinant of disease outcome in *L. infantum* visceral leishmaniasis, with relevant implications for studies on host-pathogen interaction and also for leishmanicidal drug development.

**Electronic supplementary material:**

The online version of this article (doi:10.1186/s13071-015-1259-6) contains supplementary material, which is available to authorized users.

## Background

Protozoa of the genus *Leishmania* are transmitted through the bite of phlebotomine sandflies and cause a range of human and canine diseases with varying severities. Visceral leishmaniasis, caused by *Leishmania donovani* and *L. infantum*, is the most severe of these diseases. Once symptomatic, the infection is often fatal if not treated, being responsible for the death of 20,000 to 40,000 people every year. In certain areas, such as East Africa, devastating epidemics of visceral leishmaniasis occurred recently, with very high mortality rates. Moreover, leishmaniasis are endemic in almost 100 countries around the world and HIV infection increases susceptibility to visceral disease [1].

Interestingly, not all individuals infected with visceralizing species of *Leishmania* develop pathology [2]. In their mammalian hosts, *Leishmania* reside and proliferate mainly inside macrophages. Successful control of *Leishmania* parasites is believed to require the differentiation of type 1 T helper cells, capable of producing IFNγ and allowing a “classical” activation of macrophages, enhancing their antimicrobial capacity [2]. As incomplete or inappropriate activation of this immune response will result in disease, the observed differences in pathology have been attributed mostly to host factors. However, a few previous studies have suggested some variability among *Leishmania* isolates [3–6]. Such variability may also have a high impact on disease outcome, since different strains may differ in resistance to the host’s effector mechanisms or in their capacity to activate infected macrophages.

Activated macrophages can eliminate pathogens by different means, including their exposure to highly toxic oxygen species such as superoxide, nitric oxide and hydroperoxide [7, 8]. Phagocyte oxidase (phox) and nitric oxide synthase 2 (NOS2), the enzymes responsible for the generation of superoxide (O_2_•^**−**^) and nitric oxide (NO•), respectively, are thus among the most important antimicrobial systems of macrophages [7–10]. Both enzymes have been shown to be fundamental for the control of cutaneous forms of leishmaniasis, namely in the context of mouse experimental infection with *L. major* [11, 12]. However, their relevance for the control of infections caused by visceralizing *Leishmania* is not so clear. Two previous studies addressing the role of NADPH oxidase and NOS2 in the control of *L. donovani* replication in mouse tissues gave conflicting results [11, 13], which we can now hypothesize were due to strain variations. A better understanding of the contribution of host leishmanicidal mechanisms for the control of visceral leishmaniasis deserves further investigation, as it can have a high impact on improving the management of the disease.

In this work, we used three strains of *L. infantum* isolated from different sources in the Mediterranean region to investigate their comparative infectivity patterns in mice and how these related to the parasite’s capacity to i) influence macrophage activation, and to ii) withstand the toxicity of reactive oxygen and nitrogen species. We found a high heterogeneity among *L. infantum* strains in the tested parameters, highlighting the importance of parasite variability in determining the course of visceral leishmaniasis disease.

## Methods

### Parasites

Experiments were performed with three different strains of *L. infantum* (Table 1). One of them is the reference strain MHOM/MA/67/ITMAP263, isolated in Morocco and belonging to zymodeme MON-1 [14]. The other two strains were isolated from human patients, at Instituto de Higiene e Medicina Tropical (IHMT, Lisboa, Portugal). IMT151 belongs to the MON-1 zymodeme, while IMT202 belongs to the MON-29 zymodeme. Both strains were previously genetically characterized by microsatellite markers. IMT151 was assigned to genotype 2, the most frequently found, and IMT202 was assigned to genotype 79, a unique genotype found to be close to other non-MON-1 strains and *L. donovani* east African strains [15]. Zymodeme MON-29 has been frequently associated with cutaneous lesions, as was this case (Table 1).

**Table 1:** Characterization of *L. infantum* strains used in this work

Strain	Zymodeme	Donor	Clinical manifestation
MHOM/MA/67/ITMAP263	MON-1	Adult; HIV negative	Visceral leishmaniasis
MHOM/PT/88/IMT151	MON-1	Child; HIV negative	Visceral leishmaniasis
MHOM/PT/94/IMT202	MON-29	Adult; HIV negative	Cutaneous leishmaniasis

For each experiment, parasites were re-isolated from the spleens of infected mice. Promastigotes were differentiated from spleen amastigotes by culturing at 25 °C in complete Schneider’s medium (Sigma-Aldrich Co., St Louis, MO, USA) supplemented with 10 % heat-inactivated fetal bovine serum (iFBS), 100 U/mL penicillin, 100 μg/mL streptomycin (all from Gibco, Life Technologies, Carlsbad, CA, USA), 2 % human urine, 5 μg/mL phenol red (Sigma-Aldrich Co.) and 5 mM HEPES sodium salt (Sigma-Aldrich Co.) pH 7.4. Promastigote cultures were expanded at 25 °C, for a maximum of 5 passages, in RPMI 1640 GlutaMAX™-I medium (Gibco, Life Technologies), containing 10 % iFBS, 50 U/mL penicillin, 50 μg/mL streptomycin and 20 mM HEPES sodium salt pH 7.4. Promastigote differentiation from the exponential to the stationary phase was promoted by culture at 25 °C for 5 to 7 days without medium renovation.

### In vitro susceptibility to oxidative stress

Synchronized mid-log phase promastigotes were seeded at 10^5^/well in 96-microtiter plates. Hydrogen peroxide (H_2_O_2_), menadione or diethylaminenonoate sodium salt (NONOate) (all from Sigma-Aldrich Co.) were diluted in complete RPMI medium and added to parasite-containing wells at different concentrations (10–150 μM for H_2_O_2_,1–15 μM for menadione and 625–4000 μM for NONOate). Each concentration was tested in duplicate. After 18 h at 25 °C, 2 mM resazurin (Sigma-Aldrich Co.) was added and the cultures were incubated 72 additional hours. The concentration of resorufin, the product of reduction of resazurin, in the *Leishmania* cultures was then determined by reading the fluorescence (560 nm excitation, 590 nm emission wavelengths) in a BioTek®SynergyMx (BioTek, Winooski, VT, USA) fluorometer. Wells containing untreated parasite cultures, culture medium with each concentration of the compounds, medium alone and resazurin alone were included as controls.

The concentration of each compound that caused a fifty percent decrease of promastigotes viability (IC50) was calculated using a nonlinear regression of variable slope and four parameters in GraphPadPrism 6 (GraphPad Software Inc., La Jolla, CA, USA). Three independent assays were performed and the mean and standard deviation of the three were determined. One-way ANOVA with Bonferroni *post-hoc* test was performed to detect significant differences between parasite strains.

### Animals

Mice deficient in the p47 subunit of the NADPH oxidase complex, on a C57BL/6 background (*p47phox*^*−/−*^), were bred from a breeding pair purchased from Taconic (Lille Skensved, Denmark). Mice deficient in the nitric oxide synthase 2, on a C57BL/6 background (*Nos2*^*−/−*^), were bred from a breeding pair kindly provided by Drs. J. Mudgett, J. D. MacMicking and C. Nathan (Cornell University, New York, USA). C57BL/6 (wild-type), as well as *p47phox*^*−/−*^and *Nos2*^*−/−*^ mice were bred and housed at Instituto de Biologia Molecular e Celular (IBMC; Porto, Portugal) animal facilities under specific pathogen free conditions and fed *ad libitum*. The *p47phox*^*−/−*^ mice were administered trimethoprimsulfamethoxazole (Bactrim; 600 mg L^−1^) in the drinking water, as prophylactic treatment against bacterial infection. This treatment was ceased when infection experiments began. Animals for each experimental infection were age and sex matched. Mice were euthanized by isofluorane anesthesia followed by cervical dislocation.

### Ethics statement

All animals were handled in strict accordance with good animal practice as defined by national authorities and European legislation EEC/86/609. The experimental animal procedures were approved by the Local Animal Ethics Committee of IBMC and licensed by the Portuguese Directorate-General of Food and Veterinary Medicine (DGAV, Ministry of Agriculture, Rural Development and Fishing) with reference 520/000/000/2006.

### Macrophage infection

To obtain peritoneal macrophages, naïve female C57BL/6 mice were sacrificed and the peritoneal cavity was washed with sterile PBS. The cells were centrifuged at 1200 rpm during 10 min at 4 °C and re-suspended in DMEM medium, containing 10 % iFBS, 50 U/mL penicillin, 50 μg/mL streptomycin, 2 mM L-glutamine and 10 mM HEPES sodium salt pH 7.4 (all from Gibco, Life Technologies). The suspended cells were seeded at 4 × 10^5^ cells/well in 24-well plates and incubated at 37 °C and 5 % CO_2_ during 24 h. The promastigotes were added to the culture at an infection ratio of 10 parasites per cell. After 3 h of co-incubation, non-internalized parasites were removed by gently washing with Hank’s Balanced Salt Solution (Gibco, Life Technologies) and fresh medium was added to the cells. The percentage of infected macrophages at 16 h and 72 h was determined by Giemsa staining and microscopic inspection (Leica DM E), to control experiment variability. After 16 h or 72 h, the cells were lysed and total RNA was isolated using RNeasy mini kit (QIAGEN, Hamburg, Germany), according to the manufacturer’s protocol. Total RNA quantification was performed using a NanoDrop 1000 spectrophotometer (Thermo Scientific, Waltham MA, USA), quality was assessed by visualization in a denaturating agarose gel and 0.35 μg of each sample were converted to cDNA by VILO SuperScript kit (Invitrogen, Carlsbad, USA), according to the manufacturer’s protocol. *Arginase* and *Nos2* mRNA expression were determined by quantitative real-time RT-PCR, as described below.

### In vivo infection

For in vivo infection studies, C57BL/6 (wild-type), as well as *p47phox*^*−/−*^ and *Nos2*^*−/−*^ mice were injected in the lateral vein of the tail with 2 × 10^7^ *L. infantum* stationary promastigotes in 200 μL of phosphate buffered saline pH 7.4 (PBS). At 15 and 60 days after infection, the animals were euthanized and tissues were collected and homogenized in aseptic conditions. Parasite load in each organ was estimated using a limiting dilution approach, as described previously [16, 17]. Briefly, serial dilutions of the homogenized tissue suspensions were performed in quadruplicate. After 7 to 14 days at 25 °C, the wells were examined for viable promastigotes. The reciprocal of the highest dilution that was positive for parasites was considered to be the number of parasites per mL of suspension and was used to calculate the number of parasites per organ. Statistical analysis was carried out using GraphPad Prism 6 (GraphPad Software Inc.). For multiple group and/or time point comparisons, the one- and two-ways ANOVA tests with *post-hoc* Bonferroni multiple-comparison test were used.

### Histological analysis

Liver samples were collected from each infected mouse, by cutting similarly located and similarly sized longitudinal sections from the left side lobe. These were fixed in 3.7 % buffered formaldehyde and routinely processed for embedding in paraffin. Four micrometer tissue sections were obtained using a RM2155 Leica microtome (Leica Microsystems, Wetzlar, Germany) and were stained with hematoxylin-eosin using standard procedures. An Olympus BX50 light microscope equipped with a DP21 Olympus digital camera (Olympus, Center Valley, PA, USA) was used to examine the liver sections and obtain representative pictures.

To assess the percentage of liver area (equivalent to the relative volume) occupied by granulomatous lesions, a virtual slide microscope scanner Olympus VS110 and VS-ASW software (Olympus) were used. In each tissue section, granulomatous areas were delineated by a trained operator and measured using the microscope software. The percentage of liver area occupied by granulomatous lesions was calculated from the ratio between the total area occupied by granulomas and the total area of the whole section. The median and the range of the values obtained from the (5 to 8) mice in each experimental group was determined. Statistical analysis of the results obtained in histological analysis was performed using the software STATISTICA, version 12 for Windows (StatSoft, Inc., Tulsa, OK, USA). Due to non-homogeneity of variances, nonparametric methods were applied: Kruskal-Wallis test, followed by Mann–Whitney U test with the Bonferroni with sequential correction for the p-value, in case of significant results.

### Animal tissue RNA isolation

Liver and spleen samples were collected, snap frozen in liquid nitrogen and stored at −80 °C until further use. Total RNA was isolated from tissues with the PureLink RNA Mini Kit protocol for animal tissues (Invitrogen) with the optional on-column PureLink DNase treatment (Invitrogen), according to the manufacturer’s instructions. Total RNA quantification was performed using a NanoDrop 1000 spectrophotometer (Thermo Scientific). For all samples, 1.25 μg of each were converted to cDNA by Thermoscript™ and an oligo (dT) 20 primer (Invitrogen), for 40 min at 50 °C, according to the manufacturer’s protocol.

### Gene expression measurement using quantitative real time RT-PCR

Relative levels of arginase (*Arg1*), nitric oxide synthase 2A (*Nos2*), interferon gamma (*Ifng*), tumor necrosis factor alpha (*Tnf*), interleukin (*Il*)*-10* and *Il-12p40* mRNAs were quantified by real-time PCR analysis using an iQ5 Multicolor Real-Time PCR Detection System (Bio-Rad, Hercules CA, USA). One μL of each cDNA sample was added to a reaction mix containing 10 μLiQ SYBR Green Supermix (Bio-Rad), 8 μL of ddH_2_O and 250 nM of each primer (Additional file 1: Table S1), making a total volume of 20 μL per reaction. The cycling profile was the following: 94 °C for 3.5 min, 40 cycles of 94 °C for 30 s, 59 °C for 30 s and 72 °C for 30 s. A melting curve was generated for every PCR product to confirm the specificity of the assays and a dilution series was prepared to check the efficiency of the reactions. Hypoxanthine-guanine phosphoribosyltransferase1 (*Hprt1*) was used as the housekeeping gene. The comparative CT method (2^-ΔΔCT^ method) based on cycle threshold (CT) values for *Arg1*, *Nos2*, *Ifng*, *Tnf*, *Il-10*, *Il-12p40* and *Hprt1* was used to analyze the expression levels of each non-housekeeping gene. Statistical analysis was carried out using GraphPad Prism 6 (GraphPad Software Inc.). Data normality was checked by performing Kolmogorof-Smirnoff test and Student’s T-test was used for estimating statistical significance. Multiple comparisons were performed with One-way ANOVA and *post hoc* Tukey test. A p value of less than 0.05 was considered statistically significant.

## Results

### Impact of *L. infantum* strains on macrophage activation

**Fig. 1:**
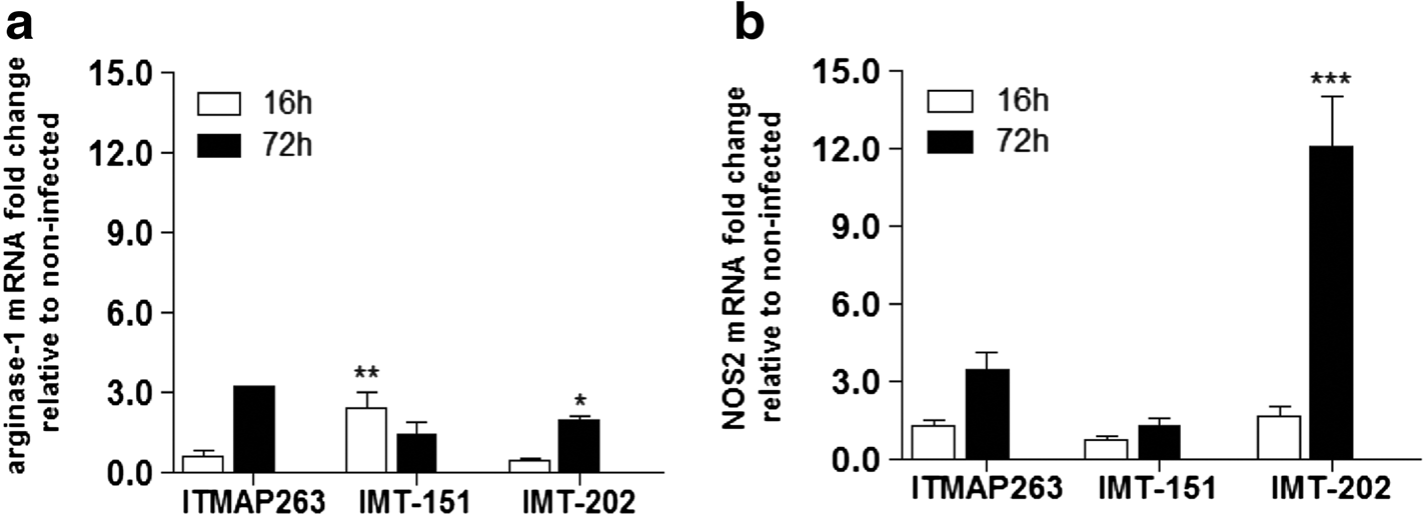
*Arg1* and *Nos2* expression profiles in macrophages infected with different *L. infantum* strains. The expression of *Arg1* (**a**) and *Nos2* (**b**) was measured by qRT-PCR in murine peritoneal macrophages after 16 and 72 h of infection with *L. infantum* ITMAP-263, IMT-151 or IMT-202 strains. Non-infected macrophages processed at the referred time points were used as control. Each sample was normalized to *Hprt* gene, calculated by the comparative C_T_ method (2^-ΔΔC^T) and the ratio between mRNA levels in infected and non-infected cells is depicted. The graph corresponds to one representative experiment out of three. Statistic analysis was performed using one-way ANOVA with Bonferroni *post-hoc* test. * *p* < 0.05; ** *p* < 0.01; *** *p* < 0.001 relative to non-infected macrophages

We started by investigating whether different *L. infantum* strains can vary in the way they activate macrophages, their natural host cell. For that purpose, peritoneal resident macrophages were collected from C57BL/6 mice and infected ex-vivo with stationary phase promastigotes of each of the following strains: the reference strain MHOM/MA/67/ITMAP263 (ITMAP-263), PT/PT/88/IMT151 (IMT-151) and MHOM/PT/94/IMT202 (IMT-202). After 16 and 72 h, the mRNA levels of *Arg1* and *Nos2* in infected macrophages were measured and compared to the levels found in non-infected macrophages cultured in parallel. As shown in Fig. 1a, at 16 h post-infection (approximately the time needed for the differentiation of promastigotes to the amastigote form), significant induction of *Arg1* was observed for the IMT-151 strain, but not the others. At 72 h, all infected macrophages showed slightly higher levels of *Arg1* expression than non-infected cells. A different picture was observed for *Nos2*. At 16 h, no significant induction was found, while at 72 h post-infection, macrophages infected with IMT-202 showed significantly increased expression of *Nos2* (about 12 times higher than non-infected macrophages), while IMT-151 and ITMAP-263 did not significantly induce this gene.

This experiment demonstrated that the different strains of *L. infantum* differed in the way they interact with macrophages, namely in the degree to which they induce the antimicrobial mechanisms of these cells.

### Expression of genes involved in host’s immune response in the livers of mice infected with different strains of *L. infantum*

**Fig. 2:**
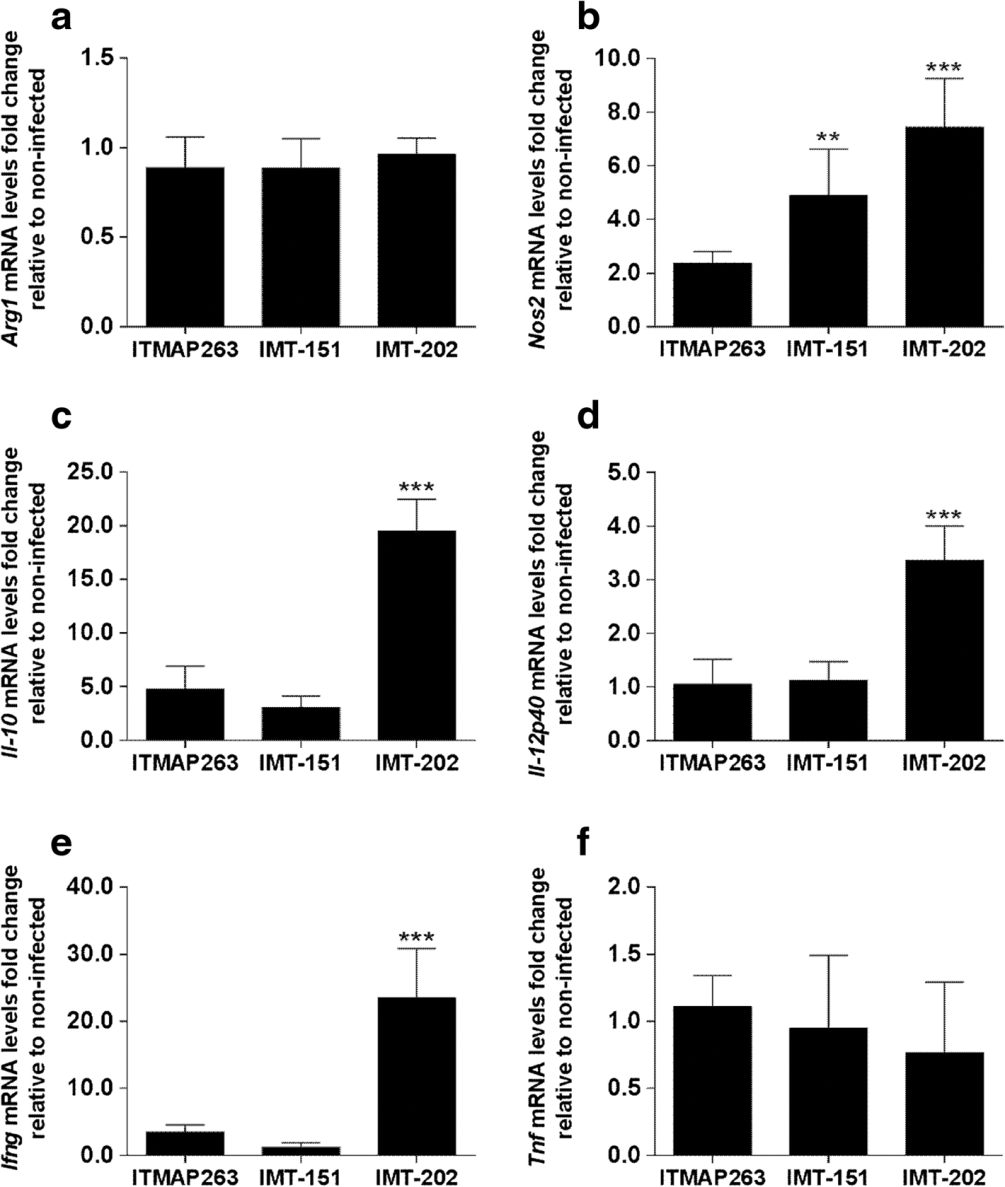
Expression profiles of genes involved in immune response in the liver of wild-type mice chronically infected with different strains of *L. infantum*. The relative expression of *Arg1* (**a**), *Nos2* (**b**), *Il-10* (**c**), *Il-12p40* (**d**), *Ifng* (**e**) and *Tnf* (**f**) in the liver of wild-type mice after 60 days of infection with *L. infantum* ITMAP-263, IMT-151 or IMT-202 strains was measured by qRT-PCR. Each sample was normalized to *Hprt*, calculated by the comparative C_T_ method (2^-ΔΔC^T). The expression was further normalized to expression levels in naïve mice’s liver. Mean and SD of each group are shown (*N* = 4–6). One-way ANOVA with *post hoc*Tukey test was performed (*p < 0.05; **p < 0.01; ***p < 0.001, versus non-infected control)

Since the three strains of *L. infantum* significantly differed in their interaction with macrophages in vitro, we investigated whether these differences were also apparent in vivo. For that, we injected intravenously C57BL/6 mice with stationary promastigotes of each *L. infantum* strain and analyzed the liver expression of *Nos2* and *Arg1* as well as of *Tnf*, *Ifng*, *Il-12p40* and *Il-10* genes, 15 and 60 days after infection. Non-infected mice of the same genetic background were used as control. Although no significant alterations in the expression of any of the genes were observed between infected and non-infected animals at day 15 post-infection (not shown), at day 60 we could readily detect different levels of *Nos2* expression. The highest expression of *Nos2* was found in the livers of mice infected with strain IMT-202 (about 8 fold higher than non-infected mice) followed by IMT-151 and ITMAP-263 (Fig. 2b). Regarding *Arg1*, no significant alterations in expression were found in any experimental condition (Fig. 2a). Interestingly, among the host immune response genes analyzed, *Il-12p40*, *Ifng* and *Il-10* were significantly upregulated only in mice infected with the strain IMT-202 (Fig. 2c-e), indicating that this strain has a higher capacity to stimulate the host immune response than the other two, triggering both pro- and anti-inflammatory signals.

### Survival and growth of different strains of *L. infantum* in mouse tissues and impact of host’s reactive oxygen and nitrogen species

In order to evaluate how the inter-strain diversity observed so far impacts on the course of in vivo infection, we infected C57BL/6 wild-type mice as well as mice deficient in the antimicrobial enzymes phagocyte NADPH oxidase (*p47phox*^−/−^) and nitric oxide synthase 2 (*Nos2*^−/−^) with each of the described *L. infantum* strains and the number of parasites in their livers and spleens were quantified after 15 and 60 days of infection.

**Fig. 3:**
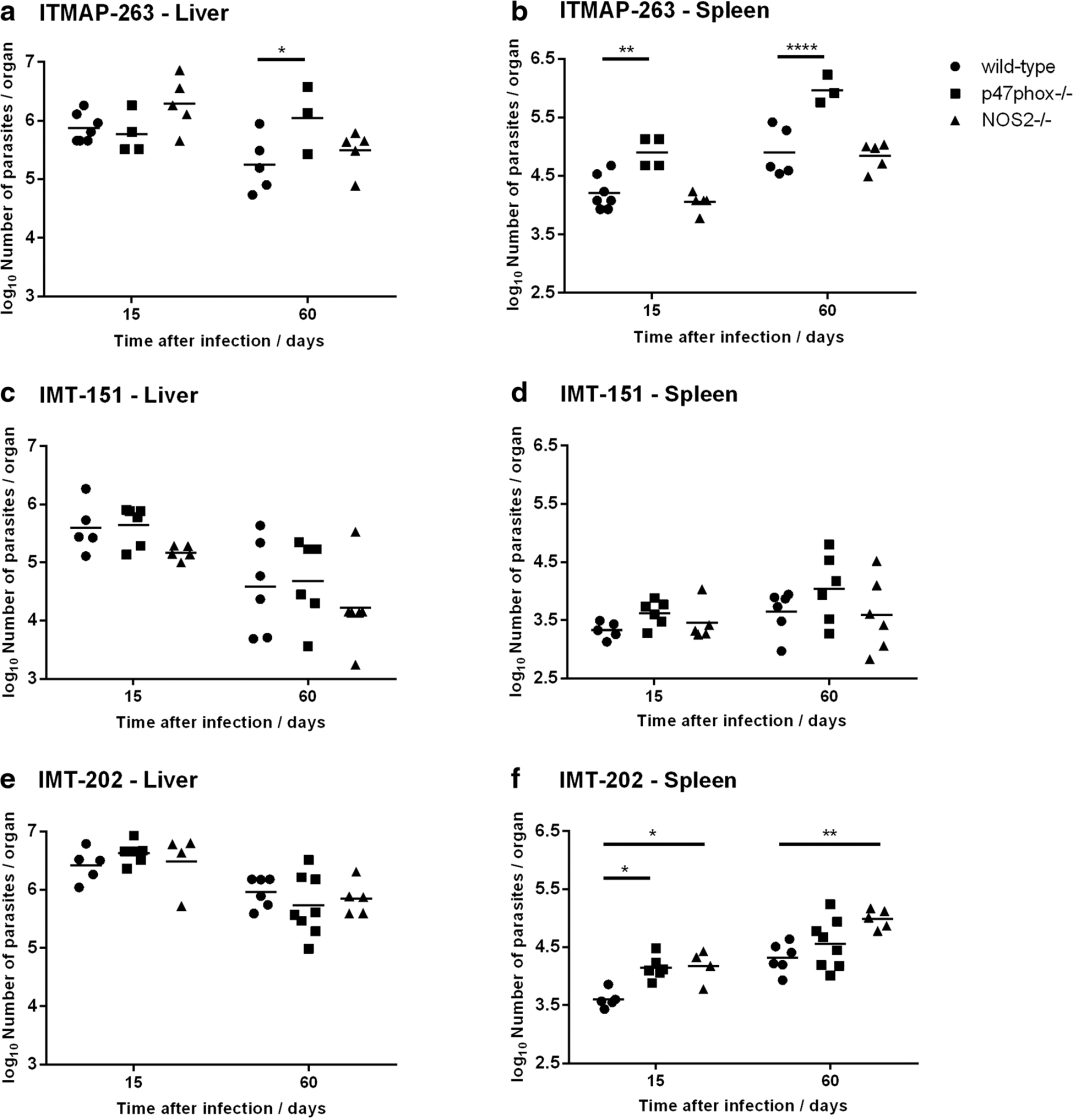
Parasite loads in wild-type, *p47phox* ^*−/−*^or *Nos2* ^*−/−*^mice infected with different strains of *L. infantum*. The parasite burden in wild-type, *p47phox* ^*−/−*^or *Nos2* ^*−/−*^ mice infected with *L. infantum* ITMAP-263 (**a**, **b**), IMT-151 (**c**, **d**) or IMT-202 (**e**, **f**) strains (liver and spleen, respectively) was determined by serial dilutions of organ homogenates at 15 and 60 days after infection. Each symbol represents one animal and the horizontal bars represent the mean log_10_ of the number of parasites per organ. Two-way ANOVA with Bonferroni *post-hoc* test was performed considering the variables “Time” and “Mouse strain”. For clarity, only statistically significant differences between mouse strains are indicated in the graphs: *p < 0.05; **p < 0.01; ****p < 0.0001

In C57BL/6 wild-type mice, each strain displayed different survival capacities in the liver, with strain IMT-151 showing the most marked decrease in parasite burden between day 15 and day 60 post-infection (1.0 log decrease on average, as compared to 0.6 and 0.5 log for ITMAP-263 and IMT-202, respectively) (Fig. 3a, c and e). In the spleen, while there was a general tendency for the increase in parasite burden between day 15 and day 60, in the case of IMT-151 this increase (0.3 log on average) did not reach statistical significance, while ITMAP-263 and IMT-202 exhibited a 0.7 log increase in spleen parasite burden in the same time interval (*p* = 0.0012 and *p* = 0.0008 between day 15 and day 60, respectively for each parasite strain) (Fig. 3b, d and f).

The three strains of *L. infantum* also differed in the way their survival and growth was affected by host’s phox and NOS2. IMT-151 parasites were partially eliminated in mouse liver and maintained its numbers in the spleen, irrespective of the expression of *p47phox* and *Nos2* (Fig. 3c and d). Parasites of the strain ITMAP-263 grew more in mice lacking *p47phox*, most notably in the spleen, but not in mice lacking *Nos2* (Fig. 3a and b). Regarding strain IMT-202, the absence of host’s *p47phox* and *Nos2* had no impact in parasite survival in the liver (Fig. 3e). However, in the spleen, the parasite burden was significantly higher in mice lacking *Nos2* both at 15 and 60 days after infection, whereas it was only transiently affected by the lack of *p47phox* (significant difference in parasite load only at day 15) (Fig. 3f).

These results showed that different isolates of *L. infantum* clearly differ in survival and growth capacity in the organs of infected mice, which may be related to the previously shown differences in their patterns of immune response stimulation. Additionally, the three strains differ in the degree to which their in vivo growth is affected by host’s phox and NOS2.

### Histological alterations in the livers of mice infected with different strains of *L. infantum*

**Fig. 4:**
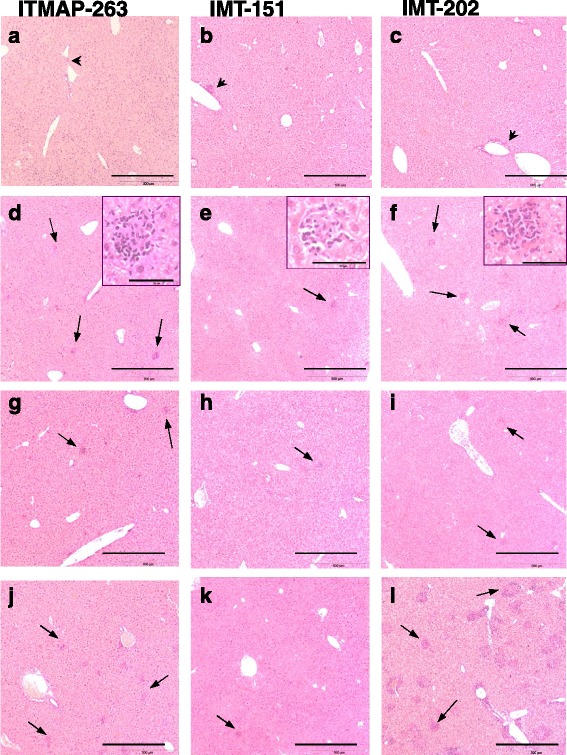
Histological alterations in the livers of mice infected with *L. infantum*. C57BL/6 (**a**-**f**), *p47phox* ^−/−^(**g**-**i**) or *Nos2* ^−/−^(**j**-**l**) mice were infected with *L. infantum* ITMAP-263 (**a**, **d**, **g**, **j**), IMT-151 (**b**, **e**, **h**, **k**) or IMT-202 (**c**, **f**, **i**, **l**). Liver sections were collected 15 (**a**-**c**) and 60 (**d**-**l**) days after infection, processed and stained with hematoxylin-eosin. Representative images of each experimental group are shown. Note the inflammatory infiltrates close to the vessels at 15 days post-infection (**a**-**c**, *blackarrow heads*) and throughout the liver parenchyma at day 60 post-infection (**d**-**l**, *black arrows*). Insets in **d**-**f** depict in a higher magnification one typical granuloma representative of each parasite strain. The bar corresponds to 500 μm, except for the insets in **d**-**f** in which the bar corresponds to 50 μm

One of the characteristics of the immune response to *Leishmania* in the liver is the infiltration of immune cells and the formation of granulomas, believed to be important for parasite containment and/or elimination [18, 19]. In order to understand whether this granuloma formation significantly differed between *L. infantum* strains, we performed the histological analysis of liver sections obtained from mice infected with each of them after 15 and 60 days of infection. The analysis was done in C57BL/6 (wild-type) as well as *p47phox*^*−/−*^ and *Nos2*^*−/−*^ mice. In Fig. 4, representative images of the most significant alterations are shown.

Fifteen days after infection, no major alterations in liver architecture were found with any of the *L. infantum* strains used, except for small lymphocytic infiltrations next to large vessels (Fig. 4a-c, arrow heads). After 60 days of infection, granulomatous lesions could be seen, typically with a macrophagic central core surrounded by lymphocytes (Fig. 4d-f, insets). The number and size of granulomas were however different between parasite strains and also between mouse genotypes (Fig. 4d-l). In order to have a clear picture of the differences in the extent of granuloma formation between experimental groups, a quantification of the granulomatous lesions was done. The results, as percentage of liver area occupied by granulomatous lesions in each situation, are shown in Table 2.

**Table 2:** Percentage of liver area occupied by granulomatous lesions in mice infected with different strains of *L. infantum* for 60 days

*L. infantum* strain	Mouse	Median	Min - Max
ITMAP-263	Wild-type	0.22	[0.09–0.59]
	*p47phox* ^*−/−*^	0.10	[0.01–0.80]
	*Nos2* ^*−/−*^	0.60	[0.27–1.22]
IMT-151	Wild-type	0.13	[0.01–0.21]
	*p47phox* ^*−/−*^	0.17	[0.02–0.44]
	*Nos2* ^*−/−*^	0.22	[0.01–0.30]
IMT-202	Wild-type	0.31	[0.18–0.55]
	*p47phox* ^*−/−*^	0.20	[0.00–0.59]
	*Nos2* ^*−/−*^	8.45	[7.62–9.90]

In C57BL/6 mice, strain IMT-202 induced the largest granuloma response, followed by strain ITMAP-263, while IMT-151 caused the lowest response. The extent of granuloma formation also varied among mouse genotypes, with *p47phox*^*−/−*^ showing a lower granuloma response and *Nos2*^*−/−*^ mice developing the largest granulomatous involvement, as compared to C57BL/6 wild-type mice. Overall, a significantly larger extent of the liver tissue was involved in granulomatous lesions in *Nos2*^*−/−*^ mice infected with *L. infantum* IMT-202 than in any other experimental group (Table 2 and Fig. 4).

### In vitro susceptibility of *L. infantum* to reactive oxygen species

Given the marked differences between strains in terms of survival and proliferation in phox- or NOS2- deficient mice, we investigated their susceptibility to reactive oxygen and nitrogen species as a possible additional factor affecting interaction with the host. Promastigotes of the three strains were cultured in vitro and exposed to different oxidative stimuli. Menadione and hydrogen peroxide (H_2_O_2_) were used as sources of reactive oxygen species and sodium nonoate was used as a nitric oxide donor. The concentration of each compound that caused a fifty percent decrease of promastigote viability (IC50) was determined and the results are shown in Table 3. ITMAP-263 showed the highest susceptibility to reactive oxygen species, having the lowest IC50 for both hydrogen peroxide and menadione, although the difference to other strains was statistically significant for menadione only. Interestingly, ITMAP-263 was significantly more resistant to sodium nonoate than the other two strains, suggesting a higher resistance to nitric oxide toxicity. No significant differences in susceptibility were found between strain IMT-151 and IMT-202.

**Table 3:** Susceptibility of promastigotes of *L. infantum* to oxidative and nitrosative stress

*L. infantum* strain	IC50 (μM)		
	H_2_O_2_	Menadione	NONOate
ITMAP-263	43 ± 11	2.6 ± 0.4	>4000
IMT-151	92 ± 15	3.9 ± 0.2 (*)	2190 ± 360 (*)
IMT-202	71 ± 29	4.7 ± 0.2 (*)	1560 ± 530 (*)

(*) *p* < 0.05, versus ITMAP-263)

Overall, these results suggest that *L. infantum* strains have significant variability, not only regarding the way they activate host’s antimicrobial mechanisms, but also the way they cope with host’s generated oxidative stress.

## Discussion

In face of a lack of bona fide virulence factors that could discriminate between high virulence and low virulence strains, *L. infantum* (and *Leishmania* species in general) have usually been considered genetically uniform and the observed range of severity in clinical manifestations of infection attributed mostly to host factors. In this work, we show that different isolates of *L. infantum* can exhibit very different infectivities, pathogenicities and resistance to host defense mechanisms.

One key aspect of *Leishmania*-host interaction is the activation of macrophage’s antimicrobial mechanisms. Mouse experimental infections with *Leishmania* contributed decisively to identify phagocyte oxidase (phox) and especially nitric oxide synthase 2 (NOS2) as fundamental antimicrobial weapons of the macrophage [20, 21]. However, while the role of NOS2 and phox in infection by *L. major* has been unequivocally shown by several authors [12, 21–23], its role in infection by visceralizing *Leishmania* species is not so clear. A first report by Murray and Nathan in 1999 demonstrated that both NADPH oxidase and NOS2 were involved in the early control of *L. donovani* replication in the liver, whereas NOS2 alone was sufficient to resolve late infection [13]. In contrast, a subsequent study showed that these antimicrobial mechanisms had no influence on the outcome of *L.donovani* infection in the liver, though NOS2 exerted moderate protective effects in the spleen at late phases of infection [11]. Among the three strains included in this work, only IMT-202 showed increased proliferation in *Nos2*^−/−^ mice, indicating once again that NOS2 is not universally involved in the control of *Leishmania* growth in mice. Interestingly, we found that NOS2 was involved in the control of IMT-202 in the spleen but not the liver, which is in line with a recent report by Nascimento et al. showing increased parasite load one week after infection in the spleens but not the livers of *Nos2*^−/−^ mice, with a strain of *L. infantum* different from those presented here [24].

We also observed that strain ITMAP-263 had increased survival in the livers of mice deficient in the phagocyte oxidase (Fig. 3a). Additionally, both ITMAP-263 and IMT-202 showed increased survival and proliferation in the spleens of phox-deficient mice, indicating that for some *L. infantum* strains phox is important for growth inhibition in this organ. Relatively few studies have evaluated the role of phox in the visceral growth of *Leishmania*, but Blos et al. have reported that it was necessary for the control of *L. major* infection in the spleen and not in other organs of mice [12].

The different in vivo infection outcomes observed in this study with three *L. infantum* strains could result either from differences in immune response activation or in resistance to the toxic reactive oxygen and nitrogen species produced by the host. Our results show that both these aspects are important and additional parasite characteristics may also play a role.

Strain IMT-151 showed a limited capacity for growth in mouse organs. Interestingly, this strain was not particularly sensitive to ROS and RNS, with IC50s for hydrogen peroxide, menadione and NONOate comparable to that of IMT-202 (Table 3) and to other values reported in the literature [25–27]. Consistent with this finding, it did not grow more in the organs of *phox*^−/−^ or *Nos2*^−/−^ mice (Fig. 3c and d). Interestingly, this strain did not significantly induce the production of pro-inflammatory cytokines in vivo which correlated with a low inflammatory response as evaluated by histological analysis. Moreover, when incubated with macrophages in vitro *L. infantum* IMT-151 led to the early induction of *Arginase* rather than *Nos2* expression, although in vivo a significant induction of *Nos2* in the liver could be seen at 60 days post-infection. Overall, the data gathered with this parasite strain indicates that it does not strongly activate the host’s immune response, but might have a low intrinsic capacity to grow in the organs of infected mice, because of defects in nutrient uptake or other survival mechanisms.

Strain IMT-202 exhibited some features of increased virulence as compared to the other two strains, both because it had a higher survival and proliferative capacity in wild-type mouse organs (Fig. 3) and because it induced a more extensive liver pathology and inflammatory response (Figs. 2 and 4 and Table 2). Of note, the IMT-202 strain was associated with the highest induction of both *Ifng* and of *Il-10* in infected mice (Fig. 2). While the first cytokine is necessary for an efficient granuloma formation [19], the second is associated with parasite persistence [19, 28].

Granuloma formation in the liver in response to *Leishmania* requires the recruitment of CD4+ T cells and the release of IL-12, IFNγ and TNF, among other cytokines [18, 19]. The proximity between IFNγ-producing T cells and infected macrophages is thought to be important for macrophage activation and parasite killing [18, 19]. Thus, granuloma formation is believed to be necessary for the containment of *Leishmania* parasites in this organ [19]. In the present work, no correlation was found between the extension of granuloma formation and the containment of parasite growth. The persistence and growth of *L. infantum* IMT-202 in spite of extensive granulomatous response could be due to the concomitant expression of *Il-10* (Fig. 2). It has been previously reported that chronic infection with visceralizing species of *Leishmania* is accompanied by the development of CD4+ T cells of the IFNγ/IL-10 double producer phenotype [28–30]. The contribution of IL-10 for parasite persistence and proliferation has been suggested to be due to decreased expression of *Nos2* in infected macrophages [28]. However, what we observed with *L. infantum* IMT-202 was concomitant expression of *Il-10* and *Nos2*. Future studies analyzing the cell types involved in cytokine production and the temporal correlation between *Nos2* and *Il-10* expression in the liver will help elucidate this apparent paradox.

It is interesting to note that *Nos2*^−/−^ mice infected with *L. infantum* IMT-202 had 30 times more liver tissue occupied by granulomas than wild-type mice infected with the same parasite strain (Table 2). This observation, suggesting an inhibitory effect of NOS2 on granuloma formation has been reported before with other pathogens [31]. Several mechanisms may contribute to explain this effect. Nitric oxide has been demonstrated to inhibit T cell proliferation and recruitment during granuloma formation [21, 32] and the activity of nitric oxide synthase may also delay granuloma formation by interfering with collagen synthesis [33].

ITMAP-263 is a reference strain, used by different investigators around the world [30, 34]. We found that this strain has a high resistance to nitric oxide (Table 3) as suggested by previous reports [34], while being more sensitive to ROS than the other two. This resistance/sensitivity profile was consistent with the growth of ITMAP-263 in the two knock-out mice models. In fact, while the parasite growth was not affected by *Nos2* expression, it was increased in the absence of *p47phox* (Fig. 3).

The high variability in sensitivity to ROS and NO found in this study suggests that field isolates vary in resistance to these antimicrobial molecules. This can have a high impact not only in their interaction with the host but also in their response to chemotherapy. In fact, several lines of research are engaged in the development of anti-leishmania drugs which act through the release of reactive oxygen or nitrogen species [35–37]. Additionally, previous reports showed a correlation between sensitivity to nitric oxide and to antimonials, both in *L. infantum* and *L. braziliensis* [34, 38], which were underlined by subtle differences in the expression of enzymes involved in basal metabolic pathways of *Leishmania* [34]. Varying resistance to antimonials has also been reported among *L. donovani* isolates, although no correlation with resistance to oxidative stress was found in this study [39]. We thus suggest that the inclusion of several parasites strains is crucial when screening new drugs for anti-*Leishmania* effect.

The “microdiversity” among isolates within a single pathogen species has been increasingly acknowledged in the case of *Mycobacterium tuberculosis*, another important human pathogen which can give rise to highly variable disease outcomes [40, 41]. In the case of *L. infantum*, although a few previous studies failed to identify major differences between strains in terms of serum sensitivity, macrophage infectivity and other markers of virulence [5, 42], a number of other reports indicate significant genetic diversity among strains [6, 15, 43]. A study done in French healthy blood donors suggested that subtle genetic differences exist between *L. infantum* parasites found in asymptomatic carriers as compared to leishmaniasis patients [4]. Moreover, a comparison between 3 isolates of *L. donovani* in India revealed different sensitivities to oxidative stress, as well as different capacities to survive and grow in mouse tissues [39]. More recently, three *L. infantum* strains isolated in Spain were shown to have distinct profiles of macrophage activation and intra-macrophagic survival [3]. In this work, we clearly show that *L. infantum* isolates differ regarding the degree to which they are controlled in vivo by both NOS2 and phox. This diversity is due both to differences in susceptibility to killing by reactive oxygen and nitrogen species and to differences in activation of the host immune response.

## Conclusions

The body of data collected in these studies allows us to conclude that it is important to increase awareness about the diversity among the agents of visceral leishmaniasis and refrain from generalizing assumptions on host-pathogen interactions and on drug effectiveness based upon studies with one or a few reference strains.

## Additional file

Additional file 1: Table S1. Primers used to amplify mouse genes. (PDF 8 kb)
