# Supplementary material for: Studies in the mouse model identify strain variability as a major determinant of disease outcome in Leishmania infantum infection
Source: Parasit Vectors. 2015 Dec 18;8:644. doi: 10.1186/s13071-015-1259-6 (PMC4684599; doi:10.1186/s13071-015-1259-6)
Supplement: Additional file 1: Table S1. — Primers used to amplify mouse genes. (PDF 8 kb) [file 13071_2015_1259_MOESM1_ESM.pdf]

Table S1. Primers used to amplify mouse genes.

Gene	Forward Primer	Reverse Primer
Il-12p40	CCTGAAGTGTGAAGCACCAA	AGTCCCTTTGGTCCAGTGTG
Il-10	CTGCTATGCTGCCTGCTCTTA	GGATCATTTCCGATAAGGCTTG
Tnf	CCGTCAGCCGATTTGCTATCT	CGGACTCCGCAAAGTCTAAG
Ifng	GAACTGGCAAAAGGATGGTGAC	GACCTGTGGGTTGTTGACCT
Nos2	ACATCGACCCGTCCACAGTAT	CAGAGGGGTAGGCTTGTCTC
Arg1	CTCCAAGCCAAAGTCCTTAGAG	GGAGCTGTCATTAGGGACATCA
Hprt	GGTGGAGATGATCTCTCAAC	TCATTATAGTCAAGGGCATATCC
